# Recommended procedures for managing carious lesions in primary teeth with pulp involvement—a scoping review

**DOI:** 10.1038/s41405-024-00259-8

**Published:** 2024-09-18

**Authors:** Ilze Maldupa, Waraf Al-Yaseen, Julius Giese, Rokaia Ahmed Elagami, Daniela Prócida Raggio

**Affiliations:** 1 Department of Conservative Dentistry and Oral Health, Riga Stradiņš University Faculty of Dentistry, Riga Stradiņš University, Rīga, Latvia; 2 School of Dentistry, College of Biomedical and Life Sciences, Cardiff University, Cardiff, Wales UK; 3 Department of Orthodontics and Paediatric Dentistry, School of Dentistry, University of São Paulo, São Paulo, Brazil

**Keywords:** Paediatric dentistry, Dentistry

## Abstract

**Background:**

Managing dental caries in primary teeth with pulp involvement is a significant challenge. Clinical guidelines offer recommendations for effective management.

**Aim:**

To identify and analyze policies, guidelines, and recommendations for treating primary teeth with pulp-involved carious lesions, highlighting existing research gaps and setting the foundation for future research.

**Methods:**

A comprehensive search was conducted across databases (PubMed, Scopus, Embase, GIN, and LILACS) and grey literature sources (Trip and ProQuest) to identify guidelines, consensus, policy, and position statements on primary teeth pulp therapy and extraction thresholds. Two independent reviewers screened the abstracts and titles, followed by full-text screening.

**Results:**

After removing duplication, of the 1098 records, 14 were selected for analysis. This review examined various treatments for deep caries lesions in primary teeth, including indirect/direct pulp capping, pulpotomy, pulpectomy, lesion sterilization/tissue restoration, and extraction. Time search was restricted to documents published from 30th January 2008 to 30th January 2024, offering insights into evolving clinical practices.

**Conclusion:**

Treatment for carious lesions in primary teeth involving the pulp depends on clinical indications and may involve minimally invasive techniques. Recommended options are indirect pulp capping, pulpotomy, and pulpectomy, while direct capping and tooth removal are discouraged. Further research is needed to address gaps, improve guideline development, and enhance consistency of recommendations.

## Introduction

Dental caries remains a significant oral health issue affecting approximately half of the global child population, with little change in prevalence over the past few decades (Uribe, Innes, & Maldupa, 2021). Despite efforts to address the problem, complications arising from carries, including pulp damage, continue to impact a considerable number of children (Lin et al., 2019). Pulp damage resulting from caries lesions accounts for nearly 90% of cases and requires effective management strategies [1].

Primary teeth present unique challenges in the treatment of pulp pathology due to their distinct anatomical and physiological characteristics, as well as the psycho-emotional development of young children [2]. While the preservation of pulp for as long as possible is advocated as the primary approach, minimally intervention dental (MID) techniques have emerged as successful preventive measures for pulp pathologies [3]. However, there are clinical scenarios where MID cannot be applied, and immediate treatment becomes necessary for pulp-related complications.

Management of carious lesion with pulp involvement in primary teeth include pulpotomy, pulpectomy, or extraction. The choice of treatment and medications is influenced by various factors. However, pulp therapy treatments often require a child’s cooperation and may involve multiple sessions or general anaesthesia. These factors can heighten anxiety for the child and increase the time and resource burden for their family and dental staff [4]. Besides, the success rate of endodontic treatment for primary teeth remains a contentious issue [5]. Consequently, extractions are increasingly favoured as the preferred treatment for pulpal pathology [4]. Nevertheless, concerns arise regarding space loss, potential orthodontic needs, and the overall impact on the child’s quality of life following premature loss of primary teeth [4].

To guide healthcare providers in making well-informed decisions regarding the management of caries lesions involving the pulp in primary teeth, various authoritative sources have developed clinical practice guidelines (CPGs), consensus statements, policy documents, and position statements. These resources aim to provide evidence-based recommendations and standardised clinical practices. However, there is considerable variability in the recommendations due to differences in available evidence, contextual factors, and variations in healthcare systems across different countries.

Numerous systematic reviews have synthesized the efficacy of pulp therapy for primary teeth, including Cochrane reviews and other comprehensive analyses [6, 7]. These reviews have significantly contributed to our understanding of the outcomes associated with different treatment approaches. Moreover, research has explored the factors influencing clinicians’ decision-making process when choosing between endodontic treatment and extraction for primary teeth with pulp involvement [8].

Despite these existing systematic reviews and research efforts, the optimal course of action for the preservation or extraction of primary teeth with pulp involvement remains a subject of ongoing debate. CPGs and recommendations have been developed to offer clear and evidence-based guidance. However, the variability in recommendations, based on the best available evidence and contextual factors, underscores the need for further exploration and examination of the existing documents related to the management of caries lesions reaching the pulp in primary teeth.

Hence, this scoping review aims to identify and describe documents (current CPGs, consensus, policies, clinical recommendations and position statements) for managing caries lesions that reached the pulp in primary teeth.

## Methods

### Protocol registration

The research protocol for this scoping review was registered on the Open Science Framework (OSF) platform to ensure transparency and adherence to the planned methodology (OSF registration: (10.17605/OSF.IO/APCKG)). The review followed the established methodology for scoping reviews outlined by the Joanna Briggs Institute (JBI) and the report followed the Preferred Reporting Items for Systematic Reviews and Meta-Analyses (PRISMA) Guidelines for Scoping Reviews [9].

### Identification of relevant studies

A comprehensive search strategy was developed in collaboration with experienced researchers and information specialists to identify relevant studies and sources of evidence. Electronic databases, including MEDLINE, Scopus, Embase, GIN, and LILACS, were systematically searched up to 30^th^ January 2024. Grey literature sources, such as Trip and ProQuest, were also consulted to capture unpublished documents, reports, and guidelines. The search terms and keywords were carefully selected to cover the key concepts of paediatric dentistry, dental caries, clinical practice guidelines, extraction, and pulp therapy (See Appendix 1).

### Inclusion criteria

This study included recent clinical practice guidelines (CPGs), consensus statements, policy documents, and position statements addressing the threshold for pulp therapy and extraction in primary teeth with pulp involvement. Eligible documents had to be officially endorsed or produced by reputable organisations like government agencies, professional associations, or expert panels, using a systematic consensus approach. In the case of multiple versions, only the latest one was considered. Time search was restricted to the last 20 years (30th January 2004) as older guidelines are likely to be updated or irrelevant. No language restrictions were applied in the search strategy.

### Exclusion criteria

Primary and secondary research articles, expert opinions, editorials, and letters to editors, as well as studies involving adult participants or special needs children.

### Study selection process

A two-step screening process was conducted by two independent reviewers. In the initial step, titles and abstracts of the identified records were screened to assess their eligibility based on the predefined inclusion and exclusion criteria. Full-text articles were obtained for potentially relevant sources, and two reviewers independently evaluated them for final inclusion in the review. Discrepancies or disagreements between the reviewers were resolved through discussion or consultation with a third reviewer to ensure consensus.

### Data extraction

Data extraction was performed using an ad hoc standardised form developed specifically for this scoping review. Two reviewers independently extracted relevant information from the included studies and documents, including publication year, authorship, study design, participant characteristics, methodology, key recommendations, and additional findings deemed relevant. Consistency and accuracy of the extracted data were ensured through cross-checking, and any disagreements were resolved through discussion or involvement of a third reviewer.

### Data analysis and presentation

Data analysis aimed to capture the range of recommendations and approaches related to the threshold for pulp therapy and extraction in primary teeth with pulp involvement. A narrative synthesis approach was employed to analyse and present the extracted data. Key themes, concepts, and recommendations from the included studies and documents were systematically organised and summarised. The extracted data were presented using table and graphs to enhance clarity and facilitate understanding. Patterns and variations among the recommendations were identified to provide a comprehensive overview of the existing literature.

## Results

### Included studies and documents

**Fig. 1:**
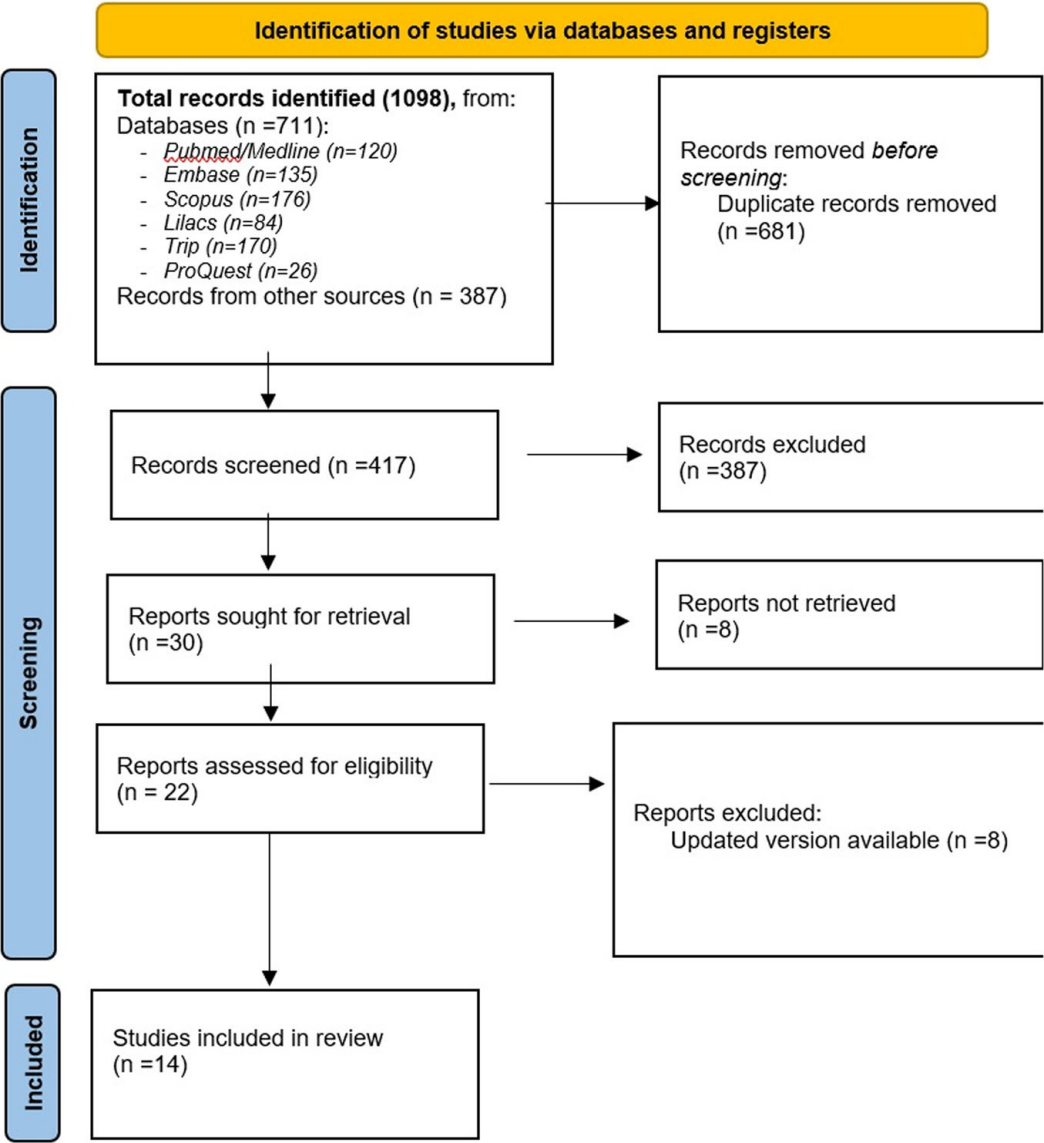
The PRISMA study flow diagram of the studies and guidelines included in this review (*n* = 14). The process consists of four stages: Identification, Screening, Eligibility, and Inclusion. Identification: a total of 1098 records were identified from various databases, including PubMed/Medline (*n* = 120), Embase (*n* = 135), Scopus (*n* = 176), Lilacs (*n* = 84), Trip (*n* = 170), ProQuest (*n* = 26), and other sources (*n* = 387). After removing 681 duplicate records, 417 records were screened. Screening: out of the 417 records screened, 387 were excluded based on title and abstract screening, and 30 reports were sought for retrieval. Eligibility: among the 30 reports, 8 were not retrieved. The remaining 22 reports were assessed for eligibility, with 8 reports being excluded due to updated versions being available. Inclusion: ultimately, 14 studies were included in the final review.

The initial search process yielded a total of 1098 records from multiple databases and organisations [American Academy of Paediatric Dentistry (AAPD), European Academy of Paediatric Dentistry (EAPD), Scottish Dental Clinical Effectiveness Programme (SDCEP), Qatari, Saudi, Health Ministry of Chile, Brazilian and Iraqi Dental Association] (See Appendix 3). After removing duplicates, the titles and abstracts of 417 unique records were screened for eligibility. From this initial screening, 30 papers were selected for a more detailed evaluation. Finally, 14 papers met the inclusion criteria and were included in the in-depth analysis. Figure 1 provides a visual representation of the study selection process following PRISMA-ScR guidelines (See Appendix 2) [10].

### Characteristics of included studies and documents

The included studies were primarily sourced from reputable databases, with the highest number of records obtained from the American Academy of Paediatric Dentistry (AAPD) (345), Scopus (176), Trip (170), Embase (135), and PubMed/MEDLINE (120). Additional sources, such as Lilacs (84), ProQuest (26), Scottish Dental Clinical Effectiveness Programme (SDCEP) (16), European Academy of Paediatric Dentistry (EAPD) (14), and GIN (6), also contributed to the final selection of papers. The selected papers focused on the treatment of primary teeth with deep caries lesions and provided insights into extraction, pulpectomy, pulpotomy, and variations within these treatment options.

### Treatment modalities

Most CPGs discussed both Direct Pulp Capping (DPC) (*n* = 6) and Indirect Pulp Capping (IPC) (*n* = 8), pulpotomy (*n* = 10) and pulpectomy (*n* = 9) as potential treatment options for specific cases involving deep caries lesions in primary teeth. IPC and DPC techniques were outlined concerning their indications, recommended protocols, and supporting evidence. Additionally, some papers explored the concept of lesion sterilisation/tissue repair (LSTR), albeit being mentioned in only two documents. The use of LSTR for managing carious lesions reaching the pulp in primary teeth was discussed in terms of its effectiveness, limitations, and potential application.

### Variations and gaps in recommendations

While the included studies and documents shared commonalities in their recommendations, some variations were observed. These variations were often influenced by contextual factors, such as the healthcare system, resources, and available treatment options in different countries or regions. Additionally, certain aspects of the management of caries lesions in primary teeth with pulp involvement lacked clear consensus or had limited evidence, indicating gaps in the existing literature.

### Summary of key findings

The reviewed literature explored various treatment options for primary teeth with deep caries lesions, including extraction, pulpectomy, pulpotomy, and their subdivisions.

### Indirect pulp capping

**Fig. 2:**
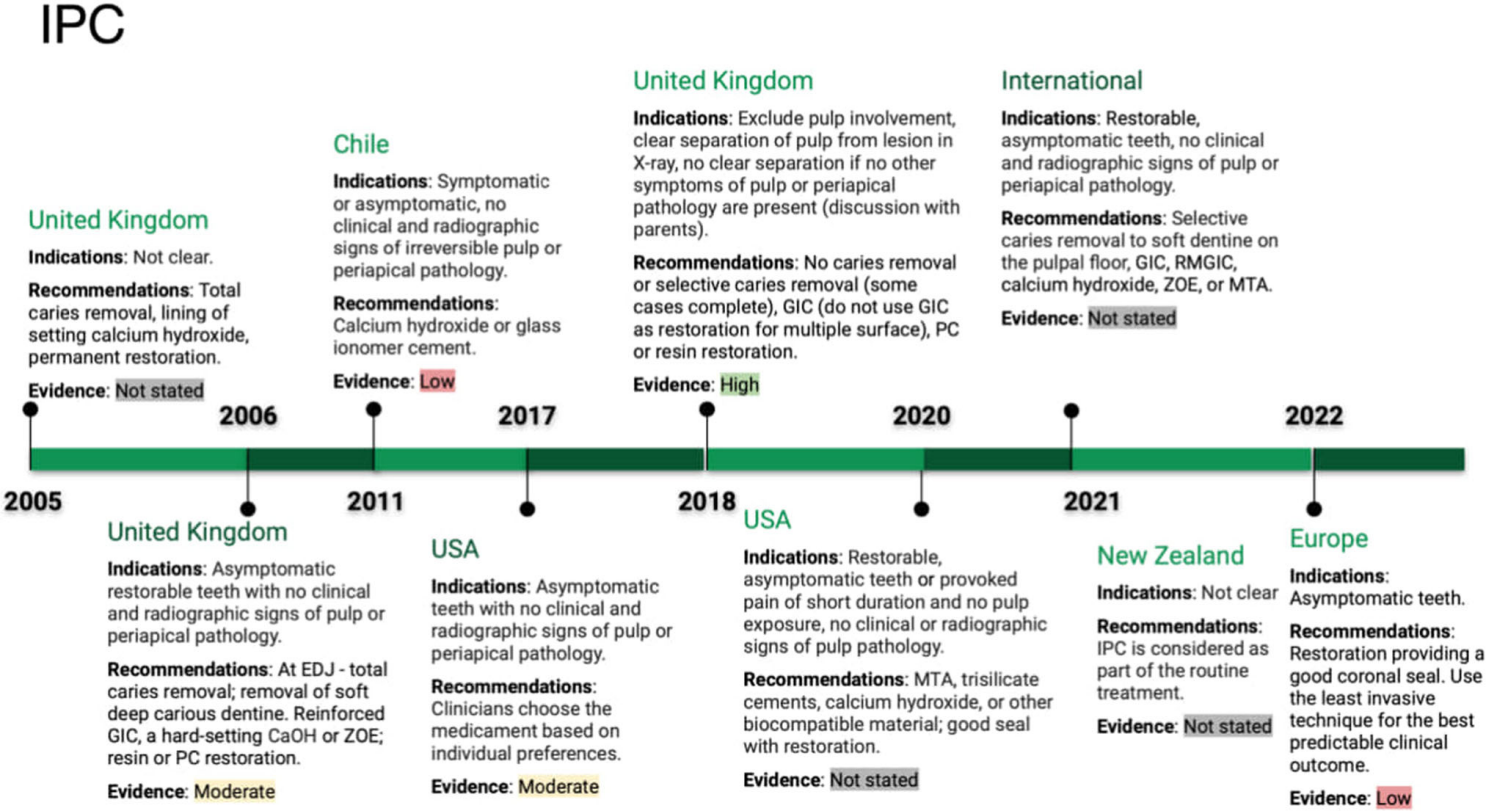
Timeline of Indirect Pulp Capping (IPC) indications and recommendations. This timeline summarises the evolution of IPC guidelines across various countries and regions from 2005 to 2022. It outlines the indications, recommendations, and levels of evidence in countries such as the UK, Chile, USA, New Zealand, and others. Specific recommendations range from total caries removal to selective caries removal with materials like calcium hydroxide and glass ionomer cement. Evidence levels vary from low to high across different years and regions.

Indirect Pulp Capping was recommended as a successful treatment for vital deciduous teeth affected by deep caries, as it is a MID approach that does not interfere with the natural exfoliation process [11, 12]. This treatment is indicated when there is no pulp involvement [13] and is considered a standard treatment option [14]. To ensure successful IPC, it is crucial to achieve an excellent seal of the coronal part of the tooth [12]. The procedure involves selective removal of soft caries tissue, particularly from the dentin-enamel junction, using hand instruments [11, 15, 16]. Subsequently, materials such as zinc oxide eugenol (ZOE), hard-setting calcium hydroxide (Ca(OH)₂), or resin-modified glass ionomer cement (RMGIC) are placed and covered with a preformed crown or adhesive restoration. These procedures have received a grade B recommendation, and level III evidence, and have shown success rates of over 90% after three years [16]. Alternative approaches, including the use of slow rotary instruments and other biocompatible materials like mineral trioxide aggregate (MTA), were also mentioned (See Fig. 2). Compared to pulpotomy treatments, IPC has demonstrated higher long-term success rates [11, 15]. Meta-analyses did not find significant differences between bonding agent liners and Ca(OH)₂, with moderate and low evidence after 24–48 months [17]. In contrast, recommendations from 2005 suggested complete removal of caries and direct pulp capping (DPC) or pulpotomy in cases of iatrogenic pulp exposure due to higher symptom occurrence and uncertain outcomes. Before filling the cavity, Ca(OH)₂ is placed to promote secondary dentin formation (See Fig. 2) [18].

### Direct pulp capping (DPC)

**Fig. 3:**
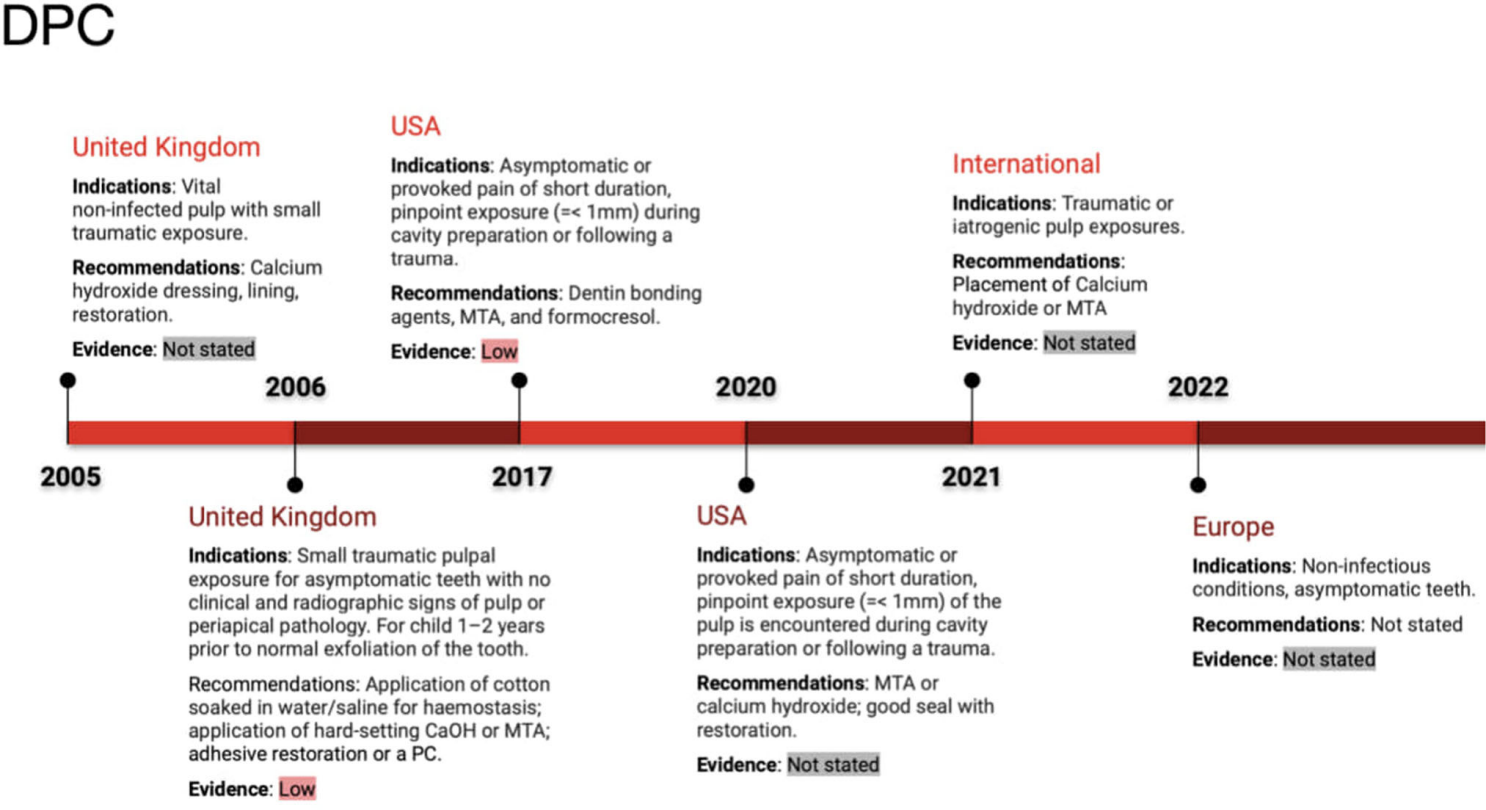
Timeline of Direct Pulp Capping (DPC) indications and recommendations. The figure displays DPC guidelines across regions from 2005 to 2022, including the UK, USA, and international recommendations. Indications include traumatic pulp exposures and materials recommended include calcium hydroxide and MTA. Evidence levels range from low to not stated, reflecting the evolving clinical recommendations over the years.

The use of DPC as a treatment option is restricted to spot-like pulpal exposure areas due to trauma or mechanical opening during caries removal in cases of non-symptomatic and non-infectious circumstances (See Fig. 3), to facilitate dentine structure development [16, 18, 19].

For the purpose of teeth protection from microleakage, Ca(OH)₂ and mineral trioxide aggregate (MTA) have been suggested [11]. Meta-analyses show no significant difference in success between Ca(OH)₂ and MTA, formocresol (FC) and dentin bonding agents after 24 months. Due to the missing discrepancy of included studies, the quality of evidence was rated as very low [17]. Prior haemorrhage control by a piece of cotton damped with saline or water has been recommended with grade C and evidence quality level IV [16].

In general, DPC is not recommended as a regular treatment option for primary teeth [15]. This might be connected to the elevated cellular density in the pulp tissue of deciduous teeth and poor prognosis [20, 21] However, close to the physiological exfoliation time, DPC can be indicated due to less severe consequences (See Fig. 3) [16].

### Pulpotomy

**Fig. 4:**
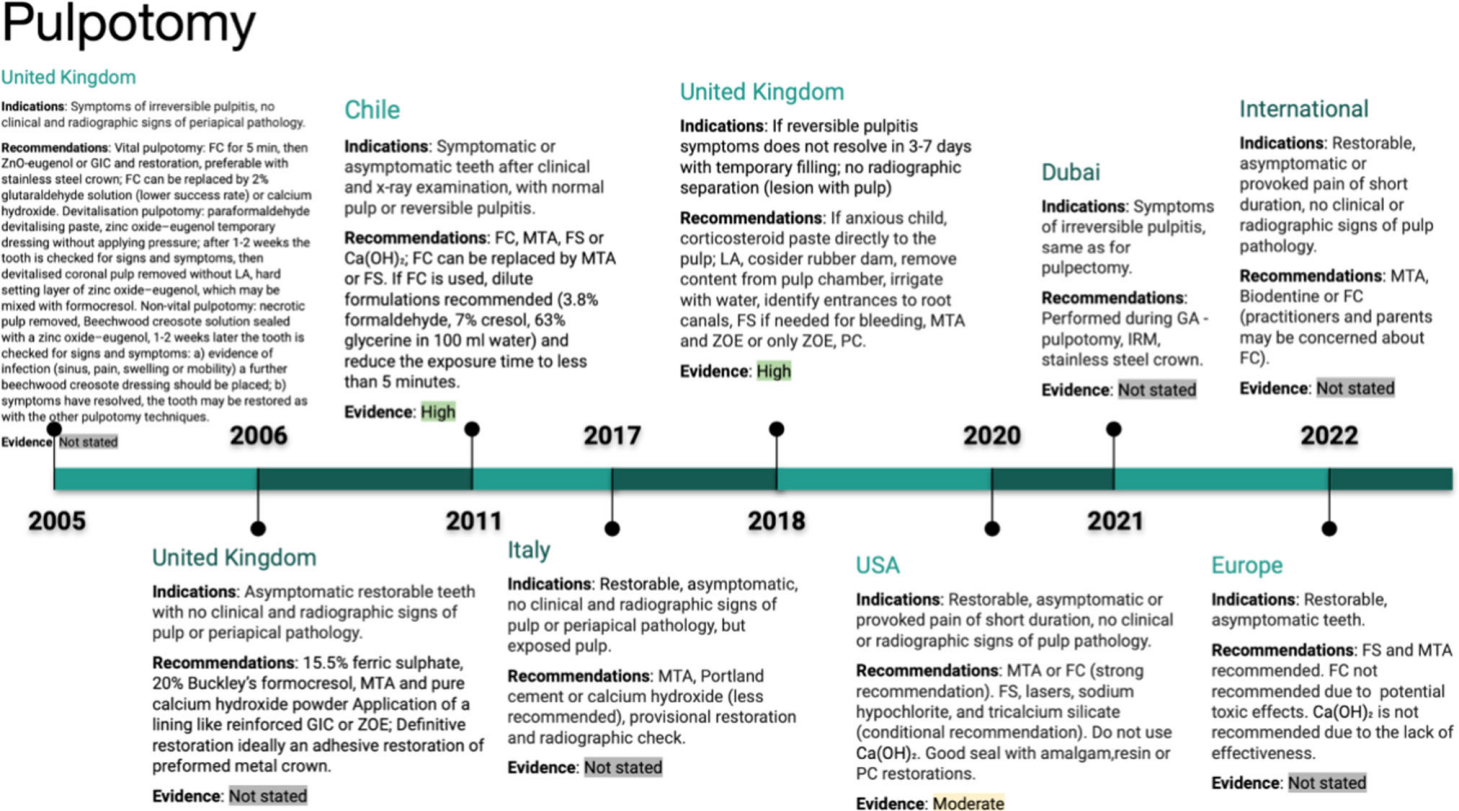
Timeline of pulpotomy indications and recommendations. This figure highlights pulpotomy guidelines in the UK, Chile, Italy, and other regions from 2005 to 2022. Indications include symptoms of irreversible pulpitis, and materials recommended include formocresol (FC), MTA, and ferric sulfate. Evidence is rated from low to high depending on the year and location. The timeline also covers regional variations in managing pulpotomy procedures.

Pulpotomy is a recommended treatment option for primary teeth with profound carious lesions, boasting a 24-month success rate of 82.6% [22]. However, due to limited direct comparisons, no definitive evidence-supported recommendation can be made regarding the choice between pulpotomy, DPC, and IPC (See Fig. 4) [14, 22].

Pulpotomy is generally indicated for primary teeth with exposed vital pulp or irreversible pulpitis of the coronal pulp, if the underlying tissue is healthy or shows reversible inflammation [16, 17]. It can be performed on deciduous teeth at any developmental stage [13]. Contraindications include severe root resorption, facial cellulitis, abscess history, or specific patient conditions necessitating general anaesthesia [23]. Pulpotomy for vital pulp in primary molars is a recommended treatment, while non-vital pulpotomy, which differs in procedure and indication, is considered obsolete in most current guidelines.

Some guidelines discourage the use of Ca(OH)₂ during pulpotomy due to compromised results and lower success rates compared to ferric sulphate (FS), mineral trioxide aggregate (MTA), and formocresol [12, 16, 17, 19].

MTA (87.8%) and formocresol (85%) have shown the highest success rates among recommended treatment choices, leading to a strong recommendation for their use [17]. Other options are conditionally recommended, and the use of formocresol may raise concerns among parents [15–17].

MTA, despite higher initial costs, proves to be more cost-effective in the long run due to its greater success rates and reduced need for secondary treatments compared to Ca(OH)₂ [17]. MTA preserves pulp integrity, reduces inflammation, and promotes tissue formation, while Portland cement is considered a low-cost alternative [24].

Additional research is needed to determine specific recommendations for lining materials, caution is advised regarding the combination of FS and eugenol, and control of haemorrhage is essential during treatment [16, 18, 24].

Stainless steel crowns are recommended as a permanent restoration after pulpotomy, while composite resin and amalgam can be used for deciduous teeth with minor structural damage [11].

### Pulpectomy

Pulpectomy is a recommended treatment for restorable primary teeth with necrosis, irreversible pulpitis, root resorption, and other pathologies [11]. It is preferred over LSTR in the absence of root resorption. Pulpectomy is generally not recommended as a first-line treatment for deep caries in vital primary molars due to the effectiveness of more conservative alternatives like indirect pulp capping or pulpotomy. However, it may be used instead of extraction when tooth loss could harm dental health and long-term occlusion, or if there is no permanent successor [12].

Prior to treatment, a periapical radiograph is taken for diagnosis, and anaesthesia is administered [4]. Root canal shaping can be done with rotary or hand files, followed by irrigation using sodium hypochlorite or alternative solutions [11, 15]. Canals are dried before using zinc oxide eugenol (ZOE) cement or calcium hydroxide (Ca(OH)₂) with iodoform paste for obturation [11, 15].

Different approaches exist for pulpectomy depending on the condition, such as two-stage or one-stage procedures [18]. The Italian Ministry of Health recommends pulpectomy for non-vital primary teeth in specific developmental stages and with clinical signs like abscesses, fistula, and pain [2]. The use of Ca(OH)₂ combined with iodoform paste is advantageous, although ZOE is also suggested [2]. Irrigation should be performed using hypochlorite, saline, or chlorhexidine [16].

**Fig. 5:**
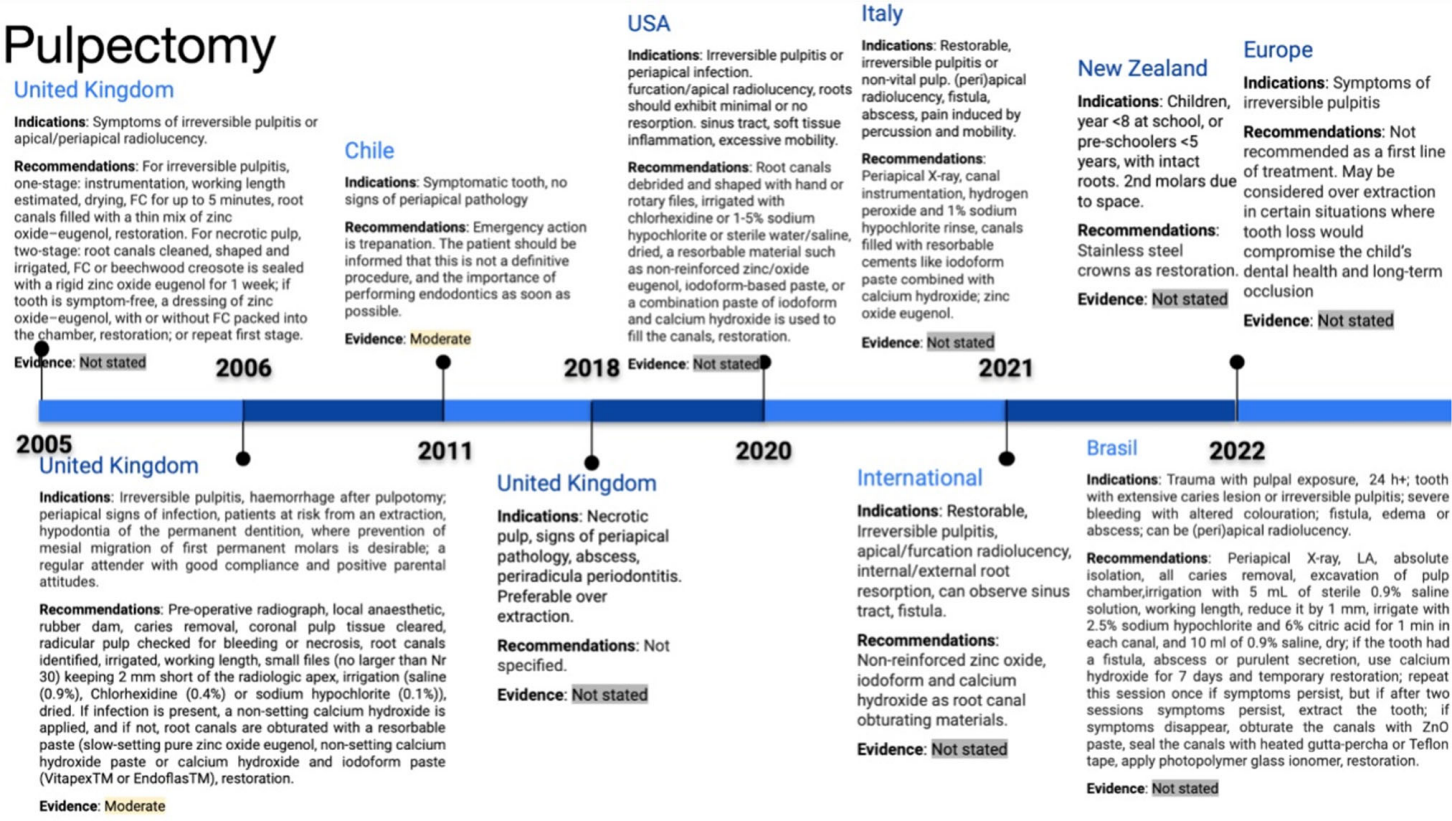
Timeline of pulpectomy indications and recommendations. The pulpectomy timeline from 2005 to 2022 illustrates recommendations from the UK, Chile, USA, and other countries. It covers the management of irreversible pulpitis and related pathology, with recommendations including the use of zinc oxideeugenol, MTA, and root canal instrumentation. Evidence levels range from low to moderate across different regions.

The Federal University of Rio de Janeiro (UFRJ) recommends specific irrigation techniques and materials for obturation, such as ZOE, glass ionomer cement (GIC), or heated gutta-percha. The heated gutta-percha is used specifically to seal the canal orifice, not to fill the canals [25]. Preformed crowns are suggested for excellent coronal seal [16]. Pulpectomy success rates range from 59% to 69% for teeth with root resorption and 84% to 90% for those without [25]. Extraction may be necessary if fistula or abscess persists after Ca(OH)₂ [25]. The Dubai Health Authority limits pulpectomy to primary teeth with less than one-third root resorption and without facial cellulitis or abscess [23]. Considerations for pulpectomy include long-term retention of second deciduous molars and stable occlusion, with conservative treatments preferred for profound carious lesions (See Fig. 5).

### Lesion sterilisation/tissue repair (LSTR)

**Fig. 6:**
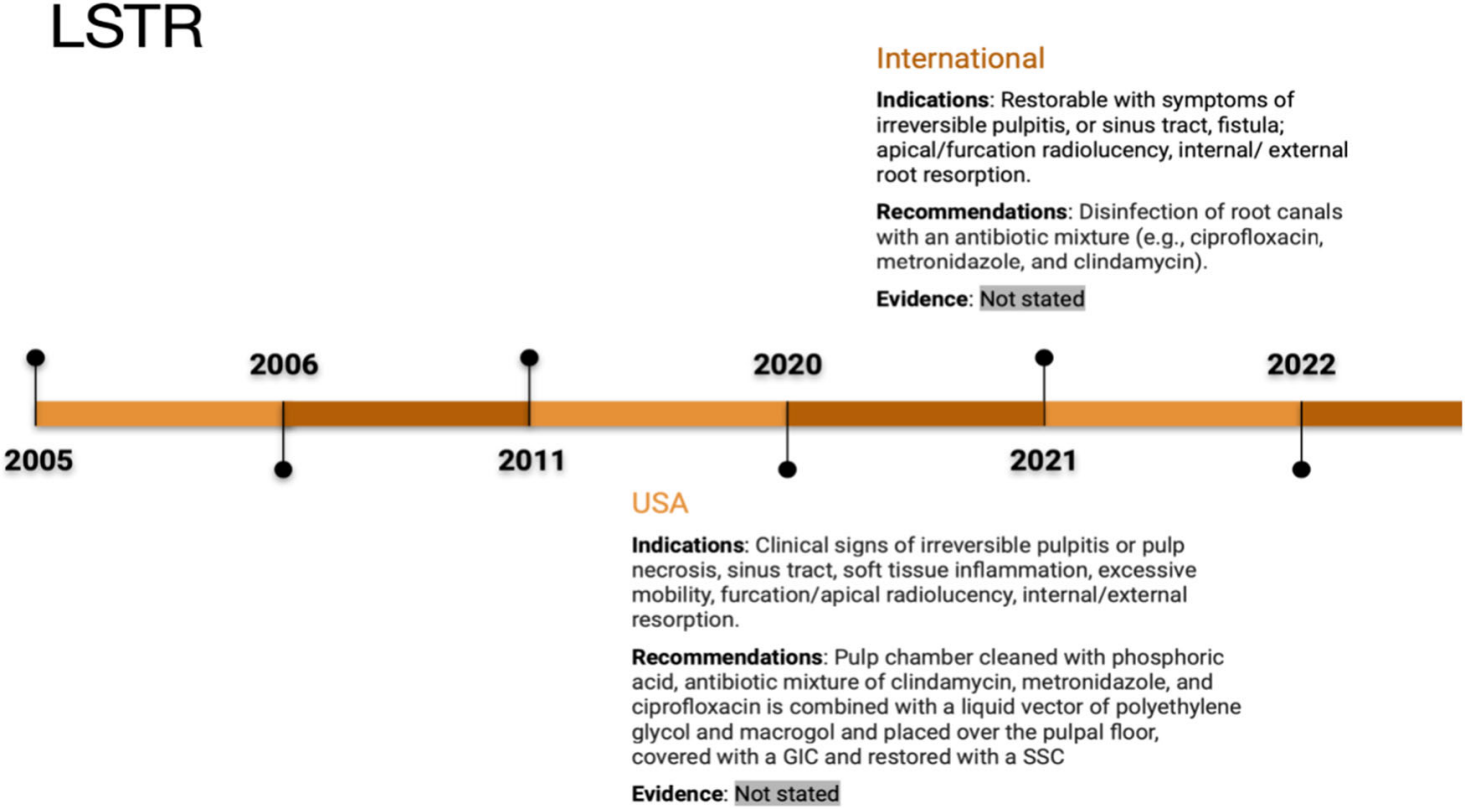
Timeline of lesion sterilisation and tissue repair (LSTR) indications and recommendations. This figure outlines LSTR treatment guidelines between 2005 and 2022 in regions like the USA and international contexts. The timeline reflects recommendations for disinfecting root canals using antibiotics like ciprofloxacin and metronidazole for cases of irreversible pulpitis and root resorption. Evidence is generally not stated.

LSTR is a possible treatment option for primary teeth experiencing clinical symptoms of irreversible pulpitis, fistula formation, and other pathologies (see Fig. 6) [11, 15]. It is considered preferable over pulpectomy in cases of root resorption and teeth expected to exfoliate within one year. The treatment involves establishing access to the pulp chamber and augmenting the orifices. Phosphoric acid is used to cleanse the chamber, followed by rinsing and drying. Subsequently, a paste containing ciprofloxacin, metronidazole, and clindamycin, along with macrogol and polyethylene, is placed in the affected areas. It is important to avoid the incorporation of tetracycline into the antibiotic mix. Finally, glass ionomer cement (GIC) and a stainless-steel crown are placed [11, 15].

### Extractions

**Fig. 7:**
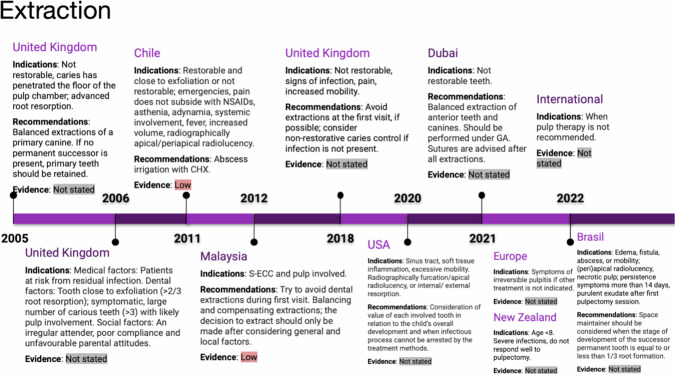
Timeline of extraction indications and recommendations. From 2005 to 2022, this figure tracks extraction guidelines in the UK, Chile, USA, and other regions. Indications include nonrestorable teeth with extensive decay or advanced root resorption. Recommendations include balanced extractions and use of chlorhexidine (CHX) irrigation. Evidence levels vary from low to not stated.

Extraction is indicated for primary teeth in the following situations: teeth approaching exfoliation, teeth that are non-restorable due to extensive caries or uncontrolled pulp haemorrhage [16, 18, 20, 26]. In addition, pulpectomy with repeated medication application without symptom relief or continuous exudation is also a reason for extraction [25]. (See Fig. 7).

Balanced bilateral extractions may be considered for primary canines, and in cases where there is absence of the contralateral tooth, extraction may be indicated for the first deciduous molars, provided that the jaw space is not excessively crowded [16, 18]. However, primary incisors are less frequently subjected to extraction [18]. It is important to consider the need for space maintainers when the development of permanent root formation does not exceed one-third of its completion [25].

In addition to clinical factors, such as tooth condition and stage of eruption, other factors including patient cooperation, social factors, and medical conditions should be considered when deciding on extraction [20]. Furthermore, the attitude of the patient and parents, as well as the number and complexity of required treatments, should be also considered [16, 18]. It is generally recommended to avoid extractions during initial dental visits [13, 20]. Whenever possible, extraction should be avoided in cases of crowding, absence of underlying permanent teeth, and situations that may cause increased stress for the patient [18].

## Discussion

The management of caries lesions that reach the pulp in primary teeth presents a complex challenge for dental professionals. CPGs play a crucial role in providing evidence-based recommendations for the treatment of such cases. This scoping review aimed to identify and describe documents, including CPGs, consensus statements, policies, and clinical recommendations, pertaining to the management of caries lesions that reached the pulp in primary teeth. Hence, this review provides valuable insights into the variations in thresholds and recommendations for different treatment procedures. Although our focus was on published documents, it is important to note that we could not verify whether these documents utilised the best available evidence to formulate their recommendations or if they had low risk of bias.

The analysis of the included documents revealed variations in thresholds and recommendations for different treatment procedures. These variations stem from differences in the interpretation of the available evidence, clinical judgement, and priorities of different dental organizations and professional societies.

The comparison of indications for each procedure among different CPGs provides valuable insights into the diverse perspectives and considerations when managing caries lesions that reach the pulp in primary teeth. These variations in recommendations reflect the complexities of clinical decision-making and the diverse approaches taken by different guidelines.

For instance, the AAPD guidelines recommend Indirect Pulp Capping as a standard treatment option for vital primary teeth with deep caries lesions but without pulp involvement [16]. IPC is widely practiced in the United States and has shown favourable outcomes. In contrast, the EAPD guidelines also support IPC but provide more specific indications, such as minimal pulp involvement and limited symptoms [12].

When considering direct pulp capping and pulpotomy, guidelines offer varying recommendations. The AAPD guidelines suggest DPC for spot-like pulpal exposures resulting from trauma or mechanical opening during caries removal [19]. On the other hand, the SDCEP guidelines discourage the routine use of DPC and instead recommend pulpotomy as a treatment option [13].

The indications for pulpotomy also show variations among guidelines. The IAPD guidelines recommend pulpotomy for primary teeth with exposed vital pulp or irreversible pulpitis of the coronal pulp [15]. However, specific indications provided by different guidelines may vary, taking into account factors such as clinical signs, developmental stages, and the prevalence of dental conditions.

Similarly, the indications for pulpectomy vary among guidelines. The AAPD guidelines recommend pulpectomy for restorable primary teeth with necrosis, irreversible pulpitis, root resorption, or other pathologies [11]. In contrast, guidelines from other sources may provide more specific indications based on their own research and clinical experience.

These variations in recommendations highlights the influence of context on treatment recommendations. Guidelines developed in different regions may reflect the specific needs and resources available in those areas. Furthermore, the availability of materials and resources can significantly impact the treatment options recommended in the guidelines. Different regions may have varying access to materials such as Ca(OH)₂, MTA, or formocresol. These variations in material availability can lead to differences in the recommended treatment modalities. Cost-effectiveness of materials play a key role in the treatment choices outlined in the guidelines. The review evidence highlights the departure from historical practices, with the more recent CPGs no longer endorsing complete caries removal [21]. Similarly, the reconsideration of calcium hydroxide (Ca(OH)₂) usage, previously employed for secondary dentin formation before cavity filling, reflects this evolving perspective. This shift aligns with our growing understanding of cariology, emphasising minimally invasive procedures to preserve teeth tissue whenever possible [27]. Despite these advancements, the adoption of these guideline points among clinicians remains limited [28]. Addressing this gap may necessitate broader dissemination of updated guidelines, targeted educational initiatives, and ongoing efforts to bridge the translation gap between evidence-based recommendations and clinical implementation.

Another important consideration is the publication and development process of the guidelines. While guidelines aim to provide evidence-based recommendations, the level of detail and transparency in their development can vary. Some guidelines may provide extensive information on the underlying evidence, the grading of recommendations, and the consensus process followed. An example of such comprehensive guidelines is the Guidelines for the Management of Dental Emergencies by the SDCEP, which provide clear explanations of the evidence base, and the consensus process used [13]. On the other hand, some guidelines may lack sufficient information on the level of evidence, or the specific studies considered during their development.The lack of transparency and detail in guideline development makes it challenging to understand the rationale behind certain recommendations and hampers the ability to compare and reconcile differences between guidelines. To enhance the transparency and quality of guidelines, future efforts should prioritize the adherence to established guideline development methodologies, such as those recommended by Guidelines International Network. This includes clearly outlining the process for evidence synthesis, the grading of recommendations, and the involvement of multidisciplinary experts. The reporting quality of clinical practice guidelines exhibits significant variability. A previous study assessing the reporting quality of CPGs in paediatric dentistry has indicated suboptimal adherence to quality standards, underscoring the need for future improvement in their applicability. Implementation of standardised checklists such as the Appraisal of Guidelines for Research and Evaluation (AGREE) is imperative during CPG development processes to ensure methodological rigour and transparency in newly developed guidelines, before their adoption into clinical practice.

Among the myriad recommended treatments for various pulp conditions, a fundamental principle must remain unequivocal—treating patients based on the minimum intervention approach. In navigating the therapeutic choices, the essence of the Hippocratic principle, “primum non nocere” (first, do no harm). Thus, as practitioners, we are reminded to balance the intricacies of diverse treatment modalities with a commitment to the overarching goal of delivering care that is both effective and minimally invasive.

## Conclusion

There are significant variations in guidelines for diagnosing and managing caries lesions that reach the pulp in primary teeth, reflecting differences in evidence interpretation, clinical judgement, and organisational priorities.

For caries with pulp involvement, guidelines differ: the AAPD advocates treating small pulpal exposures due to trauma or mechanical opening with direct pulp capping, while the SDCEP recommends more conservative approaches. The IAPD focuses on specific clinical signs and developmental stages to guide treatment, such as the extent of pulp involvement and the child’s age.

Regional needs and resources influence treatment recommendations, including the availability of materials like calcium hydroxide, mineral trioxide aggregate (MTA), or formocresol, and cost-effectiveness considerations. Recent guidelines favour minimally invasive procedures and reconsider the use of traditional materials like calcium hydroxide.

Transparency and methodological rigour in guideline development vary. Comprehensive guidelines, such as those from the SDCEP, provide clear evidence bases and consensus processes. Future guidelines should adhere to established methodologies to ensure transparency and methodological rigour.

Why this paper is important to paediatric dentists:

- There is significant variation in guidelines for managing caries in primary teeth, influenced by regional contexts, material availability, and opaque guideline development processes. Understanding this variation is crucial for paediatric dentists to interpret and apply guidelines appropriately.
- Adhering to clear guideline development methodologies and transparency about the rationale and evidence behind recommendations is needed to promote consistency and confidence in guidelines. This allows paediatric dentists to make properly evidence-based decisions.
- Enhancing the quality and usability of guidelines on managing dental caries will facilitate decision-making for paediatric dentists seeking to provide optimal patient care. Reducing ambiguity supports the provision of appropriate care tailored to specific contexts.

## Supplementary information

Appendix 1 and 2

Appendix 3

## Data Availability

The data that support the findings of this study are available in the Appendix 3.
