# Supplementary material for: Recommended procedures for managing carious lesions in primary teeth with pulp involvement—a scoping review
Source: BDJ Open. 2024 Sep 18;10:74. doi: 10.1038/s41405-024-00259-8 (PMC11410820; doi:10.1038/s41405-024-00259-8)
Supplement: Supplementary file 1 — Appendix 1 and 2 [file 41405_2024_259_MOESM1_ESM.pdf]

Appendix I: Search strategy

Database	Search query: 09/01/2024	
Pubmed/MEDLINE	("guidelines as topic"[MeSH Terms] OR "clinical practice"[Text Word] OR practice guideline*[Text Word] OR guideline*[Text Word] OR "CPG"[Text Word] OR standard*[Text Word] OR "consensus"[Text Word] OR recommendation*[Text Word] OR "statement"[Text Word] OR "Practice guidelines as topic"[Text Word] OR "best practice"[Text Word]) AND ("primary teeth"[Text Word] OR "primary tooth"[Text Word] OR "baby tooth"[Text Word] OR "deciduous dentition"[Text Word] OR "deciduous teeth"[Text Word] OR "deciduous tooth"[Text Word]) AND ("root canal therapies"[Text Word] OR "Endodontic treatments"[Text Word] OR "pulpectomy"[Text Word] OR "pulpotomy"[Text Word] OR "endodontically-treated teeth"[Text Word] OR "root canal obturation"[MeSH Terms] OR "pulp treatment" [Text Word] OR "pulp therapy" [Text Word] OR "pulpotomy" [MeSH Terms] OR "pulpectomy" [MeSH Terms] OR "endodontic therapy" [Text Word] OR "formocresol pulpotomy" [Text Word] OR "partial pulpotomy" [Text Word])	120
Embase	#1 ('guidelines as topic'/exp OR 'guidelines as topic' OR 'clinical practice'/exp OR 'clinical practice' OR 'practice'/exp OR practice AND guideline* OR guideline* OR 'cpg'/exp OR 'cpg' OR standard* OR 'consensus'/exp OR 'consensus' OR recommendation* OR 'statement' OR 'practice guidelines as topic'/exp OR 'practice guidelines as topic' OR 'best practice')<br><br>#2 ('primary teeth' OR 'primary tooth' OR 'baby tooth' OR 'deciduous dentition' OR 'deciduous teeth' OR 'deciduous tooth')<br><br>3# ('root canal therapies' OR 'endodontic treatments' OR 'pulpectomy' OR 'pulpotomy' OR 'endodontically-treated teeth' OR 'root canal obturation' OR 'pulp treatment' OR 'pulp therapy' OR 'endodontic therapy' OR 'formocresol pulpotomy' OR 'partial pulpotomy')<br><br>#1 AND #2 AND #3	135
Scopus	(TITLE-ABS-KEY("guidelines as topic" OR "clinical practice" OR "practice guideline" OR "guideline" OR "CPG" OR "standard" OR "consensus" OR "recommendation" OR "statement" OR "Practice guidelines as topic" OR "best practice")) AND (TITLE-ABS-KEY("primary teeth" OR "deciduous	176

	tooth" OR "primary tooth" OR "baby tooth" OR "deciduous dentition" OR "deciduous teeth" OR "deciduous tooth")) AND (TITLE-ABS-KEY("root canal therapies" OR "Endodontic treatments" OR "pulpectomy" OR "pulpotomy" OR "endodontically-treated teeth" OR "root canal obturation" OR "pulp treatment" OR "pulp therapy" OR "endodontic therapy" OR "formocresol pulpotomy" OR "partial pulpotomy"))	
Lilacs<br><br>https://pesquisa.bvsalud.org/portal/advanced/?lang=es<br><br>Limited to: guia de practica clinica (92)<br><br>17/02 - 84	("guidelines as topic" OR "clinical practice" OR "practice guideline" OR guideline* OR "CPG" OR standard* OR "consensus" OR recommendation* OR "statement" OR "Practice guidelines as topic" OR "best practice") AND ("primary teeth" OR "deciduous tooth" OR "primary tooth" OR "baby tooth" OR "deciduous dentition" OR "deciduous teeth" OR "deciduous tooth") AND ("root canal therapies" OR "Endodontic treatments" OR "pulpectomy" OR "pulpotomy" OR "endodontically-treated teeth" OR "root canal obturation" OR "pulp treatment" OR "pulp therapy" OR "endodontic therapy" OR "formocresol pulpotomy" OR "partial pulpotomy")	84
American Academy of Pediatric Dentistry – AAPD<br><br>1- https://www.aapd.org/search	Guideline AND pulp therapy	223
2- https://www.aapd.org/research/oral-health-policies--recommendations/	Manually - Oral Health Policies & Recommendations (The Reference Manual of Pediatric Dentistry)	122
European Academy of Paediatric Dentistry – EAPD	“Manual search”	14

https://www.eapd.eu/index.php/policies-and-guidelines		
The International Guideline Library - GIN<br><br>https://guidelines.ebmportal.com/	caries	6
TRIP - Turning Research Into Practice database<br><br>https://www.tripdatabase.com/	("guidelines as topic" OR "clinical practice" OR "practice guideline" OR guideline* OR "CPG" OR standard* OR "consensus" OR recommendation* OR "statement" OR "Practice guidelines as topic" OR "best practice") AND ("primary teeth" OR "deciduous tooth" OR "primary tooth" OR "baby tooth" OR "deciduous dentition" OR "deciduous teeth" OR "deciduous tooth") AND ("root canal therapies" OR "Endodontic treatments" OR "pulpectomy" OR "pulpotomy" OR "endodontically-treated teeth" OR "root canal obturation" OR "pulp treatment" OR "pulp therapy" OR "endodontic therapy" OR "formocresol pulpotomy" OR "partial pulpotomy")	170
Scottish Dental Clinical Effectiveness Programme – SDCEP<br><br>https://www.sdcep.org.uk/published-guidance/	"Manual search"	16
ProQuest<br><br>https://www.proquest.com/<br><br>mark (Show results outside my library's subscription.) to get all the results	noft("guidelines as topic" OR "clinical practice" OR "practice guideline" OR guideline* OR "CPG" OR standard* OR "consensus" OR recommendation* OR "statement" OR "Practice guidelines as topic" OR "best practice") AND noft("primary teeth" OR "deciduous tooth" OR "primary tooth" OR "baby tooth" OR "deciduous dentition" OR "deciduous teeth" OR "deciduous tooth") AND noft("root canal therapies" OR "Endodontic treatments" OR "pulpectomy" OR "pulpotomy" OR "endodontically-treated teeth" OR "root canal obturation" OR "pulp treatment" OR "pulp therapy" OR "endodontic therapy" OR "formocresol pulpotomy" OR "partial pulpotomy")	26

Appendix II: Scoping Review (PRISMA-ScR) Checklist

Preferred Reporting Items for Systematic reviews and Meta-Analyses extension for Scoping Reviews (PRISMA-ScR) Checklist

SECTION	ITEM	PRISMA-ScR CHECKLIST ITEM	REPORTED ON PAGE #
**TITLE**			
Title	1	Identify the report as a scoping review.	1
**ABSTRACT**			
Structured summary	2	Provide a structured summary that includes (as applicable): background, objectives, eligibility criteria, sources of evidence, charting methods, results, and conclusions that relate to the review questions and objectives.	2
**INTRODUCTION**			
Rationale	3	Describe the rationale for the review in the context of what is already known. Explain why the review questions/objectives lend themselves to a scoping review approach.	6-7
Objectives	4	Provide an explicit statement of the questions and objectives being addressed with reference to their key elements (e.g., population or participants, concepts, and context) or other relevant key elements used to conceptualize the review questions and/or objectives.	7
**METHODS**			
Protocol and registration	5	Indicate whether a review protocol exists; state if and where it can be accessed (e.g., a Web address); and if available, provide registration information, including the registration number.	11
Eligibility criteria	6	Specify characteristics of the sources of evidence used as eligibility criteria (e.g., years considered, language, and publication status), and provide a rationale.	11
Information sources*	7	Describe all information sources in the search (e.g., databases with dates of coverage and contact with authors to identify additional sources), as well as the date the most recent search was executed.	11
Search	8	Present the full electronic search strategy for at least 1 database, including any limits used, such that it could be repeated.	11
Selection of sources of evidence†	9	State the process for selecting sources of evidence (i.e., screening and eligibility) included in the scoping review.	12
Data charting process‡	10	Describe the methods of charting data from the included sources of evidence (e.g., calibrated forms or forms that have been tested by the team before their use, and whether data charting was done independently or in duplicate) and any processes for obtaining and confirming data from investigators.	12

Data items	11	List and define all variables for which data were sought and any assumptions and simplifications made.	11
Critical appraisal of individual sources of evidence§	12	If done, provide a rationale for conducting a critical appraisal of included sources of evidence; describe the methods used and how this information was used in any data synthesis (if appropriate).	-
Synthesis of results	13	Describe the methods of handling and summarising the data that were charted.	12
**RESULTS**			
Selection of sources of evidence	14	Give numbers of sources of evidence screened, assessed for eligibility, and included in the review, with reasons for exclusions at each stage, ideally using a flow diagram.	13
Characteristics of sources of evidence	15	For each source of evidence, present characteristics for which data were charted and provide the citations.	13-22
Critical appraisal within sources of evidence	16	If done, present data on critical appraisal of included sources of evidence (see item 12).	-
Results of individual sources of evidence	17	For each included source of evidence, present the relevant data that were charted that relate to the review questions and objectives.	13-22
Synthesis of results	18	Summarise and/or present the charting results as they relate to the review questions and objectives.	13-22
**DISCUSSION**			
Summary of evidence	19	Summarise the main results (including an overview of concepts, themes, and types of evidence available), link to the review questions and objectives, and consider the relevance to key groups.	23-24
Limitations	20	Discuss the limitations of the scoping review process.	23-24
Conclusions	21	Provide a general interpretation of the results with respect to the review questions and objectives, as well as potential implications and/or next steps.	25
**FUNDING**			
Funding	22	Describe sources of funding for the included sources of evidence, as well as sources of funding for the scoping review. Describe the role of the funders of the scoping review.	26

JBI = Joanna Briggs Institute; PRISMA-ScR = Preferred Reporting Items for Systematic reviews and Meta-Analyses extension for Scoping Reviews.

* Where *sources of evidence* (see second footnote) are compiled from, such as bibliographic databases, social media platforms, and Web sites.

† A more inclusive/heterogeneous term used to account for the different types of evidence or data sources (e.g., quantitative and/or qualitative research, expert opinion, and policy documents) that may be eligible in a scoping review as opposed to only studies. This is not to be confused with *information sources* (see first footnote).

‡ The frameworks by Arksey and O'Malley (6) and Levac and colleagues (7) and the JBI guidance (4, 5) refer to the process of data extraction in a scoping review as data charting.

§ The process of systematically examining research evidence to assess its validity, results, and relevance before using it to inform a decision. This term is used for items 12 and 19 instead of "risk of bias" (which is more applicable to systematic reviews of interventions) to include and acknowledge the various sources of evidence that may be used in a scoping review (e.g., quantitative and/or qualitative research,
