# Supplementary material for: Recommended procedures for managing carious lesions in primary teeth with pulp involvement—a scoping review
Source: BDJ Open. 2024 Sep 18;10:74. doi: 10.1038/s41405-024-00259-8 (PMC11410820; doi:10.1038/s41405-024-00259-8)
Supplement: Supplementary file 2 — Appendix 3 [file 41405_2024_259_MOESM2_ESM.pdf]

Appendix 3: Summary of the included Studies (n=14)

Treatment	Year	Type of document	Country	Guidelines	Diagnostics	Specific recommendations	Level of evidence	Grade of recommendation
**indirect pulp capping**	2022	Clinical practice guidelin	Europe	Duggal M, Gizani S, Albadri S, et al. Best clinical practice guidance for treating deep carious lesions in primary teeth: an EAPD policy document. Eur Arch Paediatr Dent 2022; 23: 659–666.	Asymptomatic restorable teeth with no clinical signs of pulp pathology; diagnostics and additional examination requirements not specified	Restoration providing a good coronal seal. Use the least invasive technique for the best predictable clinical outcome.	Unanimous agreement	Strong
		Clinical practice guidelin	New Zealand	Ministry of Health New Zealand. Combined Dental Agreement: Operation Guidelines for Oral Health Services for Adolescents and Special Dental Services for Children and Adolescents. Ministry of Health New Zealand, 2022.	Not clear; diagnostics and additional examination requirements not specified.	This is considered as part of the routine treatment.	Not stated	Not stated
	2021	Consensus	International	IAPD. IAPD Foundational Articles and Consensus Recommendations: Pulp Therapy for Primary and Young Permanent Teeth. IAPD, 2022.	Asymptomatic restorable teeth with no clinical and radiographical signs of pulp or periapical pathology.	Selective caries removal to soft dentine on the pulpal floor may be appropriate with deep lesions impinging on the pulp. Glass ionomer cement, resin modified glass ionomer cement, calcium hydroxide, zinc oxide/eugenol, or MTA are placed over the remaining dentine to enhance pulp healing and repair.	Not stated	Not stated
	2020	Best Practices	USA	American Academy of Pediatric Dentistry. Pulp Therapy for Primary and Immature Permanent Teeth. The Reference Manual Of Pediatric Dentistry 2020; 40: 343–351.	Asymptomatic or teeth exhibiting provoked pain of short duration relieved with over-the-counter analgesics, by brushing, or upon the removal of the stimulus. Restorable teeth with no clinical or radiographic signs of pulp pathology.	IPC can be chosen instead of DPC or pulpotomy when the pulp is normal or has a diagnosis of reversible pulpitis and there is no pulp exposure. A protective liner is a thinly-applied material placed on the dentin in proximity to the underlying pulpal surface of a deep cavity preparation, covering exposed dentin tubules to act as a protective barrier between the restorative material or cement and the pulp. Placement of a thin protective liner such as MTA, trisilicate cements, calcium hydroxide, or other biocompatible material is at the discretion of the clinician as material does not affect the IPC success. The tooth then is restored with a material that seals the tooth from microleakage.	Not stated	Not stated
	2017	Clinical practice guidelin	USA	Vineet Dhar et al. Use of Vital Pulp Therapies in Primary Teeth with Deep Caries Lesions	Asymptomatic teeth with no clinical and radiographical signs of pulp or periapical pathology.	Success is independent of the type of material used, thus clinicians choose the medicament based on individual preferences.	Low to moderate	Conditional
	2011	Clinical practice guidelin	Chile	Villanueva JM, Araneda L, Moreno JV, et al. Guía Clínica Urgencias Odontológicas Ambulatorias. Ministerio de Salud, Gobierno de Chile, 2011.	Symptomatic or asymptomatic teeth after clinical and radiographical examination, with normal pulp or reversible pulpitis.	The placement of a thin shield such as calcium hydroxide or glass ionomer cement.	Level 3 - descriptive studies	Grade C - Recommendation based on expert opinion or low quality studies only.
	2006	Clinical practice guidelin	UK	Rodd HD, Waterhouse PJ, Fuks AB, et al. Pulp therapy for primary molars. Int J Paediatr Dent 2006; 16 Suppl 1: 15–23.	Asymptomatic restorable teeth with no clinical and radiographic signs of pulp or periapical path	Local anaesthetic, good isolation with rubber dam, removal of all caries at the enamel-dentine junction, judicious removal of soft deep carious dentine (using hand excavators or a slowly rotating large round steel bur) lying directly over the pulp region with care to avoid a pulpal exposure, placement of appropriate lining material such as a reinforced glass ionomer cement, a hard-setting calcium hydroxide or zinc oxide eugenol, definitive restoration to achieve optimum external coronal seal (ideally an adhesive restoration or preformed crown).	Level III - Evidence has been obtained from a num	Grade B
	2005	Review	UK	Carrotte P. Endodontic treatment for children. Br	Not clear; diagnostics and additional examination requirements not specified.	All the carious dentine must be removed, and a thin layer of sound, non-carious dentine must remain. A lining of setting calcium hydroxide is placed, which stimulates the formation of secondary dentine. The tooth is restored over the dressing with a permanent restorative material.	Not stated	Not stated
**dirrect pulp capping**	2022	Clinical practice guidelin	Europe	Duggal M, Gizani S, Albadri S, et al. Best clinical practice guidance for treating deep carious lesions in primary teeth: an EAPD policy document. Eur Arch Paediatr Dent 2022; 23: 659–666.	Non-infectious conditions, asymptomatic teeth.	No information	Not stated	Not stated
	2021	Consensus	International	IAPD. IAPD Foundational Articles and Consensus Recommendations: Pulp Therapy for Primary and Young Permanent Teeth. IAPD, 2022.	Traumatic or iatrogenic pulp exposures.	Calcium hydroxide or MTA	Not stated	Not stated
	2020	Best Practices	USA	American Academy of Pediatric Dentistry. Pulp Therapy for Primary and Immature Permanent Teeth. The Reference Manual Of Pediatric Dentistry 2020; 40: 343–351.	Asymptomatic or teeth exhibiting provoked pain of short duration relieved with over-the-counter analgesics, by brushing, or upon the removal of the stimulus.	When a pinpoint exposure (one millimeter or less) of the pulp is encountered during cavity preparation or following a traumatic injury, a biocompatible radiopaque base such as MTA or calcium hydroxide may be placed in contact with the exposed pulp tissue. The tooth is restored with a material that seals the tooth from microleakage.	Not stated	Not stated
	2017	Clinical practice guidelin	USA	Vineet Dhar et al. Use of Vital Pulp Therapies in Primary Teeth with Deep Caries Lesions	Asymptomatic restorable teeth with no clinical or radiographic signs of pulp or periapical pathology, pulp exposure (one mm or less) encountered during caries removal.	The success of DPC was independent of the type of medicament (dentin bonding agents, MTA, and formocresol), and therefore recommends that clinicians choose the medicament based on individual preferences.	Very low	Conditional
	2006	Clinical practice guidelin	UK	Rodd HD, Waterhouse PJ, Fuks AB, et al. Pulp therapy for primary molars. Int J Paediatr Dent 2006; 16 Suppl 1: 15–23.	Small traumatic (non-carious) pulpal exposure for asymptomatic teeth with no clinical and radiographic signs of pulp or periapical pathology. Recommended for older child (1–2 years prior to normal exfoliation of the tooth) as in these cases treatment failure would not imply the need for a space maintainer following extraction, as it would in younger children.	Local anaesthetic, optimum isolation with rubber dam, gentle application of cotton pledget soaked in water/saline to stem any pulpal haemorrhage, application of hard-setting calcium hydroxide paste or mineral trioxide aggregate (MTA), definitive restoration to achieve optimum external coronal seal (ideally an adhesive restoration or preformed metal crown).	Level IV - No studies of good quality are availabl	Grade C - generally not recommended
	2005	Review	UK	Carrotte P. Endodontic treatment for children. Br	Vital non-infected pulp with small traumatic exposure.	A calcium hydroxide dressing is placed directly over the pulp, followed by a lining and restoration.	Not stated	Not stated
**Pulpotomy**	2022	Clinical practice guidelin	Europe	Duggal M, Gizani S, Albadri S, et al. Best clinical practice guidance for treating deep carious lesions in primary teeth: an EAPD policy document. Eur Arch Paediatr Dent 2022; 23: 659–666.	Asymptomatic restorable teeth with no clinical signs of pulp pathology; diagnostics and additional examination requirements not specified	Ferric Sulphate and MTA recommended. Formocresol not recommended due to it's potential toxic effects. Calcium hydroxide is not recommended due to the lack of effectiveness.	Not stated	Not clear; only strong recommendation to not to use FC.
		Clinical practice guidelin	New Zealand	Ministry of Health New Zealand. Combined Dental Agreement: Operation Guidelines for Oral Health Services for Adolescents and Special Dental Services for Children and Adolescents. Ministry of Health New Zealand, 2022.	Not clear; diagnostics and additional examination requirements not specified.	Pulpotomy should only be used in unusual cases, it is certainly not intended as a routine procedure.	Not stated	Not stated
	2021	Consensus	International	IAPD. IAPD Foundational Articles and Consensus Recommendations: Pulp Therapy for Primary and Young Permanent Teeth. IAPD, 2022.	Asymptomatic restorable teeth with no clinical and radiographical signs of pulp or periapical pathology.			
		Clinical practice guidelin	Dubai	Health Policies and Standards Department, Health Regulation Sector. Guidelines for Pediatric Dentistry. Dubai Health Authority; Government of Dubai, 2021.	Symptoms of irreversible pulpitis, but no clear information about clinical and radiograohic signs. The same indications as for pulpectomy.	Performed during GA - pulpotomy, IRM, stainless steel crown.	Not stated	Not stated
	2020	Best Practices	USA	American Academy of Pediatric Dentistry. Pulp Therapy for Primary and Immature Permanent Teeth. The Reference Manual Of Pediatric Dentistry 2020; 40: 343–351.	Asymptomatic or teeth exhibiting provoked pain of short duration relieved with over-the-counter analgesics, by brushing, or upon the removal of the stimulus. Restorable teeth with no clinical or radiographic signs of pulp pathology.	Remove coronal pulp, control pulpal hemorrhage. When the coronal tissue is amputated, the remaining radicular tissue must be judged to be vital without suppuration, purulence, necrosis, or excessive hemorrhage that cannot be controlled by a cotton pellet after several minutes. Treat remaining vital radicular pulp tissue with long-term medicament (MTA or formocresol). Other materials (ferric sulfate, lasers, sodium hypochlorite, and tricalcium silicate) have conditional recommendations. Do not use calcium hydroxide for pulpotomy. Fill pulp chamber with suitable base and seal tooth from microleakage with restoration. Use amalgam or composite resin for teeth with a lifespan of two years or less and sufficient enamel support. Use stainless steel crown for multisurface lesions.	Not stated	MTA and formcresol recommended; other medicaments - conditional recommendation.
	2017	Clinical practice guidelin	USA	Vineet Dhar et al. Use of Vital Pulp Therapies in Primary Teeth with Deep Caries Lesions	Asymptomatic teeth with no clinical and radiographical signs of pulp or periapical pathology, but exposed pulp during caries removal.	For pulpotomy, MTA, formocresol, FS, laser, NaOCl, tricalcium silicate can be used, but not calcium hydroxide.	quality evidence for FS, and laser, very low-quality evidence for NaOCl and tricalcium silicate; low-quality evidence to NOT to use calcium hydroxide. The systematic review suggests that the overall success rate at 24 months for MTA,	MTA and FC, but conditional for FS, laser, NaOCl and tricalcium silicate; conditional recommendation to NOT to use calcium hydroxide.
			Italy	Gherlone E, Allegrini S, Annibali S, et al. Clinical recommendations in odontostomatology. Ministry of Health, Italy, 2017.	Asymptomatic restorable teeth with no clinical and radiographical signs of pulp or periapical pathology, but with exposed pulp.	Complete removal of carious tooth tissue must precede pulp chamber opening to avoid bacterial contamination. During operating procedures designed to remove pulp from the chamber, it is important to use abundant irrigation to avoid damaging or overheating root pulp. Any bleeding from root openings must stop spontaneously within four minutes at most. The next step is to position the chosen material (MTA, Portland cement or calcium hydroxide (less recommended)), construct a provisional restoration and carry out a radiographic check.	Not stated	Not stated
	2011	Clinical practice guidelin	Chile	Villanueva JM, Araneda L, Moreno JV, et al. Guía Clínica Urgencias Odontológicas Ambulatorias. Ministerio de Salud, Gobierno de Chile, 2011.	Symptomatic or asymptomatic teeth after clinical and radiographical examination, with normal pulp or reversible pulpitis.	Pulpotomy is a procedure in which the chamber pulp is amputated and the root pulp is devitalised, with the aim of removing all infected or inflamed tissue while maintaining the root pulp. Formocresol, MTA, ferric sulphate or a layer of pure calcium hydroxide can be used for this purpose. FC due to toxic properties can be replaced by MTA or ferric sulphate without decreasing the likelihood of successful treatment or FC can be diluted (3.8% formaldehyde, 7% cresol, 63% glycerine in 100 ml water) and the exposure time on pulp stumps should be reduced to less than 5 minutes.	Level 1 - evidence from RCT	recommended, based on good quality studies. In interventions: systematic reviews of randomised clinical trials, randomised clinical trials, other systematic reviews with or without meta-
	2006	Clinical practice guidelin	UK	Rodd HD, Waterhouse PJ, Fuks AB, et al. Pulp therapy for primary molars. Int J Paediatr Dent 2006; 16 Suppl 1: 15–23.	Asymptomatic restorable teeth with no clinical and radiographic signs of pulp or periapical path	Local anaesthetic, Good isolation with rubber dam, Removal of caries, complete removal of roof of pulp chamber preferably with a non-end cutting bur, removal of coronal pulpal tissue with sharp sterile excavator or large round bur in a slow handpiece, attain initial radicular pulpal haemostasis by gentle application of sterile cotton pledget moistened with saline (haemostasis should be achieved within four minutes). Selection of medicament for direct application to radicular pulp stumps to include any of the following:<br>1) 15.5% ferric sulphate solution, burnished on pulp stumps with microbrush for 15 seconds to achieve haemostasis, followed by thorough rinsing and drying;<br>2) 20% (1:5 dilution) Buckley's formocresol solution applied to radicular pulp on a cotton pledget for five minutes to achieve superficial tissue fixation;<br>3) MTA paste applied over radicular pulp with proprietary carrier;	Level Ia and Ib - Evidence is available from meta	Grades A and B
	2005	Review	UK	Carrotte P. Endodontic treatment for children. Br	Symptoms of irreversible pulpitis, but no clinical and radiograohic signs of periapical pathology to perform vital or devitalization pulpotomy. If radiolucency observed periapically, non-vital pulpotomy recommended.	Can be done as vital pulpotomy (Buckley's formocresol for 5 min, then ZnO-eugenol or GIC and restoration, preferable with stainless steel crown; FC can be replaced by 2% glutaraldehyde solution (lower success rate) or calcium hydroxide (following haemostasis, calcium hydroxide powder was delivered to the pulp chamber using a small, sterile, endodontic amalgam carrier; failure of this technique is explained by the presence of an extra-pulpal clot separating the calcium hydroxide from the pulpal tissue and thus impairing healing)); as devitalisation pulpotomy (paraformaldehyde devitalising paste applied and soft layer of zinc oxide–eugenol temporary dressing is then placed, without applying pressure; after one to two weeks the tooth is checked for signs and symptoms, then devitalised coronal pulp removed without the need for local anaesthesia, hard setting layer of zinc oxide–eugenol, which may be mixed with formocresol) or as non-vital pulpotomy (necrotic pulp contents are removed as other pulpotomy and using small excavators; Beechwood creosote solution on a cotton pledget is sealed into the cavity with a zinc oxide–eugenol dressing, one to two weeks later the tooth is checked for signs and symptoms: a) evidence of infection (sinus, pain, swelling or mobility) a further beechwood creosote dressing should be placed; b) symptoms have resolved, the tooth may be restored as with the other pulpotomy techniques).	Not stated	Not stated
**Pulpectomy**	2022		Brasil	Laura Guimarães Primo et al. Protocolo De Pulpectomia Para Dentes Decíduos: Um Guia Clínico E Prático Baseado Em Evidências Científicas, 2022	Spontaneous or persistent pain or absence of pain; tooth that have suffered trauma with pulpal exposure, time interval greater than 24 hours; tooth with extensive caries lesion or unsatisfactory restoration associated with signs and symptoms of irreversible pulpitis; severe bleeding with altered colouration (no longer bright red) that does not stop after a few minutes during coronal opening. Clinically can be presence of fistula, edema or abscess; radiographically can be apical/periapical radiolucency. Contraindicated if patient has systemic condition (immunocompromised, pre or post-transplant, or with risk of infectious endocarditis).	In cases of uncooperative patient, the professional must perform the procedure in two or more sessions. Periapical X-ray, local anesthesia and absolute isolation. Remove all decayed tissue from the pulp chamber using a carbide bur or dentin excavator, then use a spherical diamond-tipped bur to drill into the pulp chamber and follow up with a tapered carbide bur. To irrigate the pulp chamber, use 5 mL of sterile 0.9% saline solution while simultaneously aspirating to visualize canal orifices. Determine working length conventionally or electronically and reduce it by 1 mm. Use 2.5% sodium hypochlorite for chemical-mechanical preparation and irrigate with 6% citric acid for 1 min in each canal to remove smear layer. Finish with a final irrigation of 10 ml of 0.9% saline and dry with absorbent paper. If the tooth had a fistula, abscess or purulent secretion, use calcium hydroxide for 7 days and temporary restoration. Repeat this session once if symptoms persist, but if after two sessions symptoms persist, extract the tooth. If symptoms disappear or if the tooth did not have any fistula, abscess or purulent secretion, obturate the canals with ZnO paste. To seal the canals, use heated gutta-percha on a sterilized stick or Teflon tape for isolation, or apply photopolymer glass ionomer. Use sterile Teflon isolation tape or gutta-percha on a stick, torch, alcohol, match, or lighter for best results. Finish with permanent resin restoration.	Not stated	Not stated
		Clinical practice guidelin	Europe	Duggal M, Gizani S, Albadri S, et al. Best clinical practice guidance for treating deep carious lesions in primary teeth: an EAPD policy document. Eur Arch Paediatr Dent 2022; 23: 659–666.	Symptoms of irreversible pulpitis	Pulpectomy is not recommended as a first line of treatment for deep caries management of vital primary molars, due to the existence of more conservative successful alternatives. Nevertheless, pulpectomy may be considered over extraction in certain situations where tooth loss would compromise the child's dental health and long-term occlusion (i.e., minimise space loss) or such as in the absence of a permanent successor.	Not stated	Not stated
		Clinical practice guidelin	New Zealand	Ministry of Health New Zealand. Combined Dental Agreement: Operation Guidelines for Oral Health Services for Adolescents and Special Dental Services for Children and Adolescents. Ministry of Health New Zealand, 2022.	Children, year 8 and under at school, or pre-schoolers under five years of age, with intact roots. Severe infections associated with primary teeth do not respond well to pulpectomy. There are occasions, although rare, where a pulpectomy is required on a deciduous second molar, in order to keep this tooth long term.	Stainless steel crowns may be placed after pulp therapy	Not stated	Not stated
	2021	Consensus	International	IAPD. IAPD Foundational Articles and Consensus Recommendations: Pulp Therapy for Primary and Young Permanent Teeth. IAPD, 2022.	Symptoms of irreversible pulpitis, restorable tooth, apical/furcational radiolucency, internal/external root resorption, clinically can observe sinus tract, fistula.	Removal of the coronal and radicular pulp followed by debridement of the root canal(s); resorbable materials such as non-reinforced zinc oxide, iodoform and calcium hydroxide as root canal obturating materials.	Not stated	Not stated
	2020	Best Practices	USA	American Academy of Pediatric Dentistry. Pulp Therapy for Primary and Immature Permanent Teeth. The Reference Manual Of Pediatric Dentistry 2020; 40: 343–351.	Symptoms of irreversible pulpitis or periapical infection. Radiographically furcation/apical radiolucency, or evidence of internal/external resorption can be observed; the roots should exhibit minimal or no resorption. Clinically could be a sinus tract, soft tissue inflammation not resulting from gingivitis or periodontitis, excessive mobility not associated with trauma or exfoliation.	The root canals are debrided and shaped with hand or rotary files and then irrigated with chlorhexidine or 1-5% sodium hypochlorite or sterile water/saline. Because it is a potent tissue irritant, sodium hypochlorite must not be extruded beyond the apex. After the canals are dried, a resorbable material such as non-reinforced zinc/oxide eugenol (ZOE), iodoform-based paste, or a combination paste of iodoform and calcium hydroxide is used to fill the canals. The tooth then is restored with a restoration that seals the tooth from microleakage.	Not stated	Not stated
			Italy	Gherlone E, Allegrini S, Annibali S, et al. Clinical recommendations in odontostomatology. Ministry of Health, Italy, 2017.	Restorable tooth, symptoms of irreversible pulpitis or non-vital pulp. Radiographically apical/periapical radiolucency, clinically fistula, abscesses, pain induced by percussion and mobility not connected to tooth shedding.	solutions diluted to 1%, resorbable cements like iodoform paste combined with calcium hydroxide; zinc oxide eugenol, which has good crown sealing capacity but limited antibacterial activity, is damaging to corresponding permanent teeth and acts as an irritant on periapical tissues; calcium hydroxide, whose action mechanism seems to be mainly linked to its properties of alkalinity and the presence of calcium ions, it is useful for necrotic teeth with extensive periapical damage; it is more quickly resorbed than zinc oxide eugenol.	Not stated	Not stated
	2011	Clinical practice guidelin	Chile	Villanueva JM, Araneda L, Moreno JV, et al. Guía Clínica Urgencias Odontológicas Ambulatorias. Ministerio de Salud, Gobierno de Chile, 2011.	Symptomatic or asymptomatic tooth, but with no signs of periapical pathology clinically or radiographically.	Emergency action is trepanation. The patient should be informed that this is not a definitive procedure, and the importance of performing the importance of performing endodontics as soon as possible.	Level 2 - Cohort studies, case-control studies, non-randomised clinical trials.	Grade B - Recommendation based on moderate quality studies.
	2006	Clinical practice guidelin	UK	Rodd HD, Waterhouse PJ, Fuks AB, et al. Pulp therapy for primary molars. Int J Paediatr Dent 2006; 16 Suppl 1: 15–23.	Symptoms of irreversible pulpitis or haemorrhage after pulpotomy; radiographically with or without periapical signs of infection. Patients at risk from an extraction (e.g. bleeding disorders, hereditary angiooedema), Patients at risk if a general anaesthetic is required for tooth removal (e.g. some cardiac conditions, cystic fibrosis, muscular dystrophies), minimal number of extensively carious primary molars likely to require pulp therapy, hypodontia of the permanent dentition, Where prevention of mesial migration of first permanent molars is desirable, A regular attender with good compliance and positive parental attitudes.	The procedure involves a one- or two-stage pulpectomy depending on whether the radicular pulp is irreversibly inflamed or non-vital. A pre-operative radiograph, local anaesthetic, rubber dam. The caries is removed, the roof of the pulp chamber is removed, and any remains of coronal pulp tissue are cleared. The radicular pulp is checked for bleeding or necrosis, and root canals are identified, irrigated, and estimated for working length. Small files (no larger than Nr 30) are used to lightly file canal walls keeping 2 mm short of the radiologic apex, and canals are irrigated (saline (0.9%), Chlorhexidine (0.4%) or sodium hypochlorite (0.1%)) and dried. If infection is present, a non-setting calcium hydroxide is applied, and if not, root canals are obturated with a resorbable paste (slow-setting pure zinc oxide eugenol, non-setting calcium hydroxide paste or calcium hydroxide and iodoform paste (VitapexTM or EndoflasTM). The procedure ends with a definitive restoration to achieve an optimum external coronal seal. To remove irreversibly inflamed or necrotic radicular pulp tissue and gently clean the root canal system; to obturate the root canals with a filling material that will resorb at the same rate as the primary tooth and be eliminated rapidly if accidentally extruded through the apex. 86% clinical success at 36 months follow up (lower success rates found at longer follow-up times).	Level IIa and IIb - Evidence is available from randomised controlled trials and other well conducted clinical studies.	Grade B

	2005	Review	UK	Carrotte P. Endodontic treatment for children. Br	Symptoms of irreversible pulpitis or apical/periapical radiolucency.	If the radicular pulp is found to be irreversibly inflamed a one-stage technique may be undertaken: the root canals are identified and instrumented to the working length estimated from a pre-operative radiograph; after drying the canals with paper points, formocresol is applied for up to 5 minutes; the root canals are then filled with a thin mix of zinc oxide–eugenol, using a rotary paste filler, and the restoration of the tooth is completed. If the radicular pulp is necrotic, a two-stage procedure is required: the root canals are again cleaned, shaped and irrigated to remove all necrotic debris; a pledget of cotton wool moistened with either formocresol or beechwood creosote is sealed in the pulp chamber with a rigid zinc oxide eugenol dressing for one week; at the subsequent visit the tooth should be symptom-free, firm, without a discharging sinus (if not, a second application of beechwood creosote is required); if the tooth is found to be symptomless, a dressing of zinc oxide–eugenol, with or without the addition of formocresol, is packed into the base of the chamber and the tooth finally restored.	Not stated	Not stated
**Lesion sterilization and tissue repair**	2021	Consensus	International	IAPD. IAPD Foundational Articles and Consensus Recommendations: Pulp Therapy for Primary and Young Permanent Teeth. IAPD, 2022.	Restorable teeth with symptoms of irreversible pulpiti, or sinus tract, fistula; radiographically sapical/furcational radiolucency, internal/ external root resorption.	Disinfection of root canals with an antibiotic mixture (e.g., ciprofloxacin, metronidazole, and clindamycin).	Not stated	Not stated
	2020	Best Practices	USA	American Academy of Pediatric Dentistry. Pulp Therapy for Primary and Immature Permanent Teeth. The Reference Manual Of Pediatric Dentistry 2020; 40: 343–351.	Symptoms of irreversible pulpitis or necrosis or a tooth treatment planned for pulpotomy in which the radicular pulp exhibits clinical signs of irreversible pulpitis or pulp necrosis. Clinically a sinus tract, soft tissue inflammation not resulting from gingivitis or periodontitis, excessive mobility not associated with trauma or exfoliation can be observed. Radiographically furcation/apical radiolucency, or radiographic evidence of internal/external resorption.	After opening the pulp chamber of a necrotic tooth, the canal orifices are enlarged using a large round bur to create medication receptacles. The walls of the chamber are cleaned with phosphoric acid and then rinsed and dried. A three antibiotic mixture of clindamycin, metronidazole, and ciprofloxacin is combined with a liquid vector of polyethylene glycol and macrogol to form a paste placed directly into the medication receptables and over the pulpal floor.60 It then is covered with a glass-ionomer cement and restored with a stainless steel crown.	Not stated	Not stated
**Extraction**	2022		Brasil	Laura Guimarães Primo et al. Protocolo De Pulpectomia Para Dentes Decíduos: Um Guia Clínico E Prático Baseado Em Evidências Científicas, 2022	Clinically no symptoms or edema, fistula, abscess, or mobility; radiographically apical/periapical radiolucency, necrotic pulp. In cases of persistence of symptoms for more than 14 days: if after the second application of intracanal medication in necrotic pulp treatmemt, and there is no regression of signs and symptoms and the tooth continues with purulent exudate.	Space maintainer should be considered when the stage of development of the successor permanent tooth is equal to or less than 1/3 root formation.	Not stated	Not stated
		Clinical practice guidelines	Europe	Duggal M, Gizani S, Albadri S, et al. Best clinical practice guidance for treating deep carious lesions in primary teeth: an EAPD policy document. Eur Arch Paediatr Dent 2022; 23: 659–666.	Symptoms of irreversible pulpitis if other treatment is not indicated.	Not relevant	Not stated	Not stated
		Clinical practice guidelines	New Zealand	Ministry of Health New Zealand. Combined Dental Agreement: Operation Guidelines for Oral Health Services for Adolescents and Special Dental Services for Children and Adolescents. Ministry of Health New Zealand, 2022.	Children, year 8 and under at school, or pre-schoolers under five years of age, with intact roots. Severe infections associated with primary teeth do not respond well to pulpectomy. There are occasions, although rare, where a pulpectomy is required on a deciduous second molar, in order to keep this tooth long term.			
	2021	Consensus	International	IAPD. IAPD Foundational Articles and Consensus Recommendations: Pulp Therapy for Primary and Young Permanent Teeth. IAPD, 2022.	If pulp therapy is not recommended, then extraction should be considered.	Not relevant	Not stated	Not stated
		Clinical practice guidelines	Dubai	Health Policies and Standards Department, Health Regulation Sector. Guidelines for Pediatric Dentistry. Dubai Health Authority; Government of Dubai, 2021.	All non-restorable teeth. Balanced extraction of anterior teeth especially canines must be considered.	Extractions should be performed under GA. Sutures are advised after all extractions, surgicel can be used in combination with sutures in cases of persistent bleeding and/or children with coagulation disorders.	Not stated	Not stated
	2020	Best Practices	USA	American Academy of Pediatric Dentistry. Pulp Therapy for Primary and Immature Permanent Teeth. The Reference Manual Of Pediatric Dentistry 2020; 40: 343–351.	Consideration of value of each involved tooth in relation to the child's overall development and when infectious process cannot be arrested by the treatment methods. Clinically a sinus tract, soft tissue inflam- mation not resulting from gingivitis or periodontitis, excessive mobility not associated with trauma or exfoliation. Radiographically furcation/ apical radiolucency, or radiographic evidence of internal/ external resorption.	Not relevant	Not stated	Not stated
			Italy	Gherlone E, Allegrini S, Annibali S, et al. Clinical recommendations in odontostomatology. Ministry of Health, Italy, 2017.	Thorough diagnosis is necessary to establish whether the tooth must be avulsed if the conditions for performing correct endodontic treatment are not met. Not restorable, indications not clear.	Not relevant	Not stated	Not stated
	2012	Clinical practice guidelines	Malaysia	Medical Development Division of Malaysia. Management Of Severe Early Childhood Caries. CPG Secretariat Health Technology Assessment Section Medical Development Division, 2012.	Extraction of primary teeth is one of the treatment options in managing children with S-ECC although the clinician should try to avoid dental extractions during the child's first visit. For teeth that are pulpally involved, the clinician may decide to conduct endodontic treatment or extraction. The decision to extract should only be made after considering both general factors (patient's cooperation, medical condition, dental infection - may increase patient's morbidity) and local factors (restorability, extent of caries which may involve the pulp and roots, potential for malocclusion of disturbances in development of the dentition - balancing and compensating extractions may be considered).	Not relevant	Level III, Opinions or respected authorities, based on clinical experience; descriptive studies and case reports; or reports of expert committees	Not stated
	2011	Clinical practice guidelines	Chile	Villanueva JM, Araneda L, Moreno JV, et al. Guía Clínica Urgencias Odontológicas Ambulatorias. Ministerio de Salud, Gobierno de Chile, 2011.	Restorable AND close to exfoliation or not restorable; emergencies (localised pain in the vestibular fundus, constant, severe, sustained, spontaneous, lancinating, pulsating type that increases with palpation in the vestibular fundus or in the palatine or lingual area. Sensation of a long tooth, which occludes earlier in the dental arch. Pain does not subside with NSAIDs. Asthenia, adynamia. Systemic involvement. Feverish sensation. Increase in volume. (described symptoms for each specific diagnosis); radiographically apical/periapical radiolucency.	As a result of pulp necrosis, there is an absence of irrigation in the pulp tissue, and for the same reason, in the tissues surrounding an abscess, antibiotics are not effective and are considered adjuvant therapy to dental and/or surgical treatment. Use CHX irrigation.	Level 3-4 (descriptive studies and expert opinions); for recommnedations of CHX rinse, level of evidence 1, grade of recommendation A.	Grade C - Recommendation based solely on expert opinion or low quality studies.
	2006	Clinical practice guidelines	UK	Rodd HD, Waterhouse PJ, Fuks AB, et al. Pulp therapy for primary molars. Int J Paediatr Dent 2006; 16 Suppl 1: 15–23.	Medical factors: Patients at risk from residual infection (e.g. immunocompromised, susceptibility to infective endocarditis). Dental factors: Tooth close to exfoliation (>2/3 root resorption); contralateral tooth already lost (in the case of a first primary molar, and if indicated orthodontically), not restorable; the presence of any intra-oral swelling or sinus; a history of intra-oral or facial swelling; extensive internal root resorption; symptomatic large number of carious teeth with likely pulpal involvement (>3). Social factors: An irregular attender, with poor compliance and unfavourable parental attitudes.	Not relevant	Not stated	Not stated
	2005	Review	UK	Carrotte P. Endodontic treatment for children. Br	Grossly decayed teeth; not restorable even after pulp therapy; in teeth where caries has penetrated the floor of the pulp chamber; in teeth with advanced root resorption, or those close to exfoliation. Balanced extractions are rarely justified for primary incisors. The loss of a primary canine, however, may have a significant effect on the arch and balanced extractions should always be considered.Ppoorly cared for dentition requiring multiple treatments, the complex conservation of one tooth in the presence of a number of comparable teeth of doubtful prognosis is poor paediatric dentistry and should be avoided. Avoided wherever possible in certain groups of children; ie those with bleeding disorders, or medical conditions such as diabetes where general anaesthesia is contraindicated. Primary teeth should also be retained where a radiograph reveals the lack of a permanent successor.	Not relevant	Not stated	Not stated
